# Supplementary figures and images for: Enhanced bacteriostasis and osseointegrative properties of SiRNA-modified polyetheretherketone surface for implant applications
Source: PLoS One. 2024 Dec 5;19(12):e0314091. doi: 10.1371/journal.pone.0314091 (PMC11620434; doi:10.1371/journal.pone.0314091)

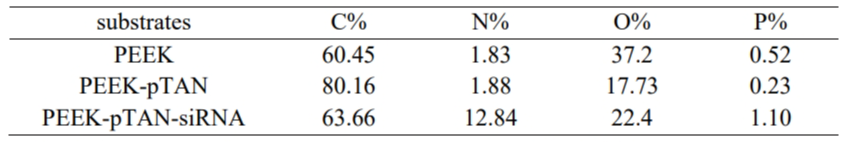

Supplement: S1 Table — Percentage calculations were based on the quantities of C, N, O and P only. (TIF) [file pone.0314091.s001.tif]

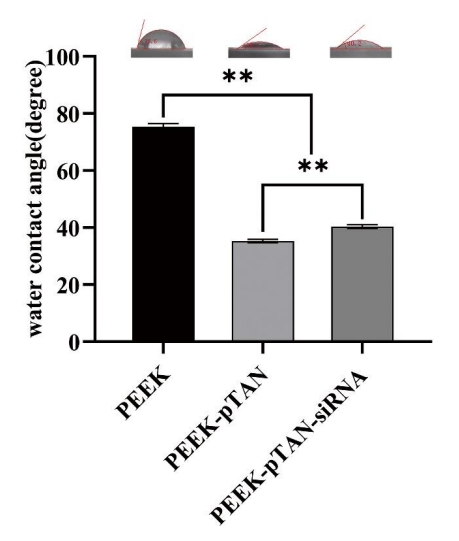

Supplement: S1 Fig — (TIF) [file pone.0314091.s002.tif]

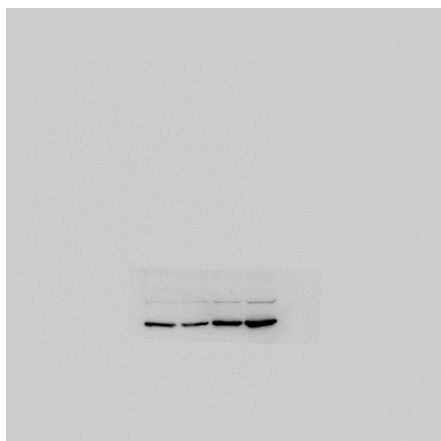

BMP-2

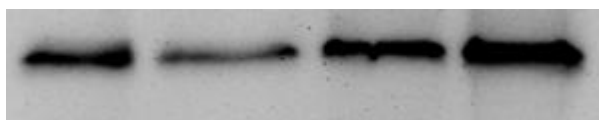

ALP

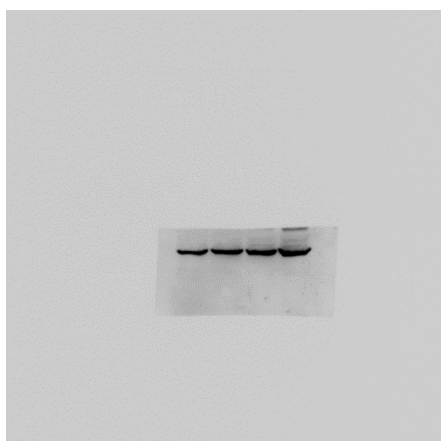

RUNX2

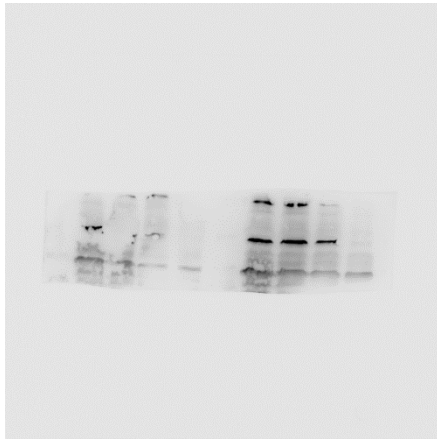

CKIP-1

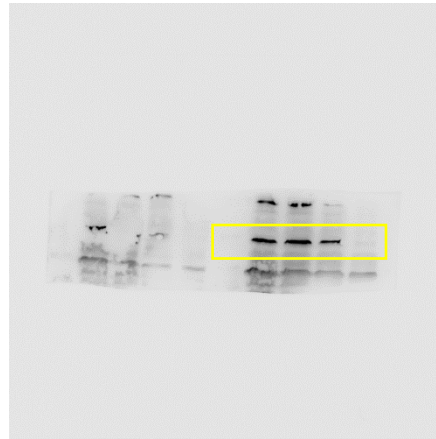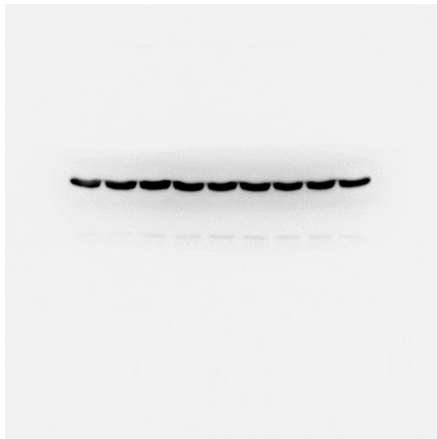

GAPDH

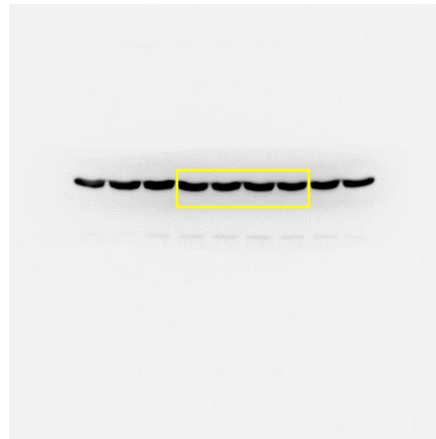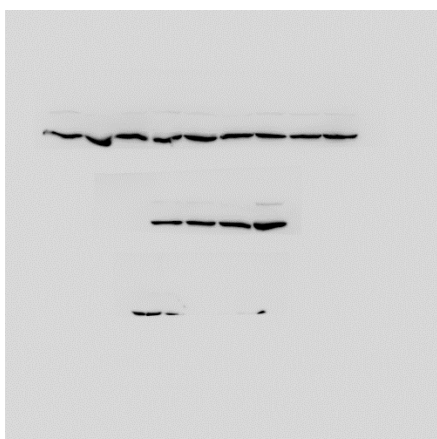

pSmad1/5

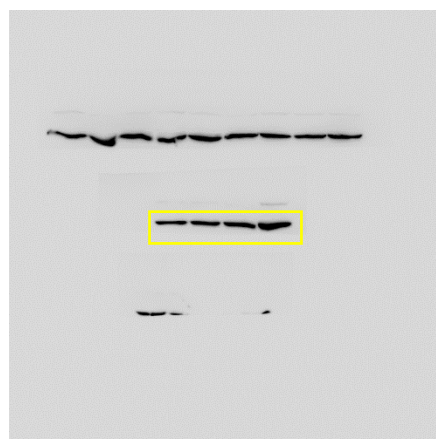

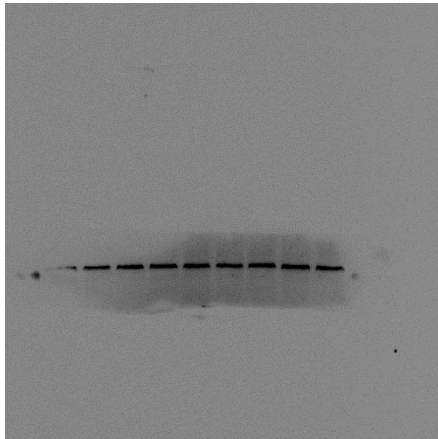

Smad1/5

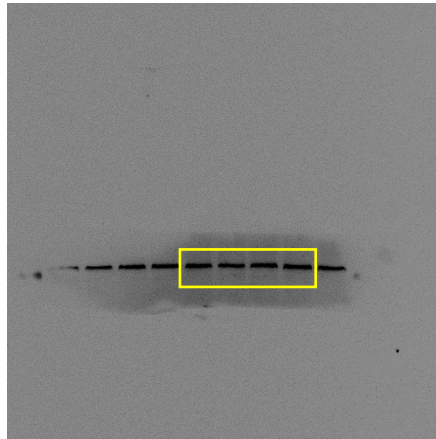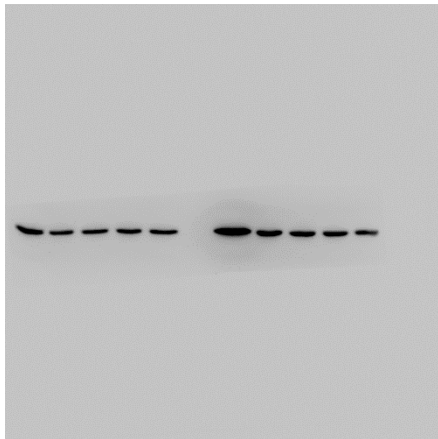

GAPDH

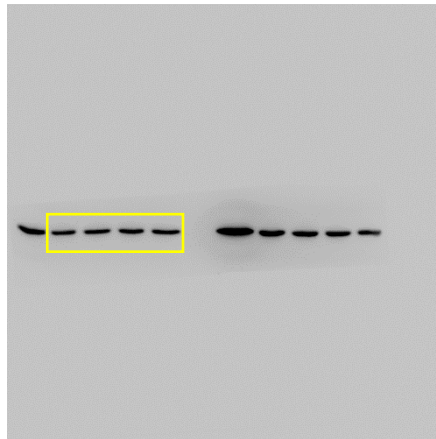

Supplement: S1 Raw images — (PDF) [file pone.0314091.s004.pdf]
