# Supplementary material for: Enhanced bacteriostasis and osseointegrative properties of SiRNA-modified polyetheretherketone surface for implant applications
Source: PLoS One. 2024 Dec 5;19(12):e0314091. doi: 10.1371/journal.pone.0314091 (PMC11620434; doi:10.1371/journal.pone.0314091)
Supplement: Attachment — Submitted filename: Review of PLOS one paper.docx [file pone.0314091.s005.docx]

**Peer Review**

Enhanced Bacteriostasis and Osseointegrative Properties of siRNA Modified Polyetheretherketone Surface for Implant Applications

**Summary:**

In recent years, polyetheretherketone (PEEK) has gained popularity as an implant material in clinical applications due to its biocompatibility, chemical stability, radiolucency, and elastic modulus similar to that of natural bone. However, PEEK faces challenges such as poor integration with surrounding bone tissue and susceptibility to bacterial infection. To enhance the bio-functional activity of PEEK, authors coated its surface with an adhesive layer of pTAN, which possesses antibacterial properties, enabling the subsequent immobilization of the PEI/siRNA complex. The modified surface demonstrated improved osseointegration both in vivo and in vitro, as well as inhibition of various bacterial infections in vitro assays. Additionally, they showcased the osteogenic potential of the modified PEEK surface in vivo,

**General comments:**

The manuscript addresses a significant topic in bone implantation and presents an efficient approach for modifying the surface of PEEK-implants, thus providing experimental evidence to support its clinical application. The methodology is sound and manuscript is clearly written, well-structured with clear research questions and a logical flow, however there are several areas including data interpretation, which require improvement to enhance the clarity and impact of the study.

**Major Concerns**

1. In Fig 1B, please provide Immunofluorescence images of control groups as well and explain the observation in the manuscript text. In Fig 1C, kindly mention whether you are reporting your surface roughness in Ra or Rq. Also, the AFM images seem to be hiding those numbers mentioned on top of each image. Additionally, please include more detailed analysis as to why you obtained those numbers and how they relate to the surface roughness of all three groups. For Fig 1D, you mentioned getting boost in N1s signal in PEEK-pTAN-siRNA surface. Could you provide expected atomic concentration in %? It would have been lot more helpful if the data was tabulated from XPS spectra.
2. In Fig 2 we see a lot less red fluorescence for PEEK-pTAN and PEEK-pTAN-siRNA groups. Do authors have other images showing better red fluorescence? Or the reason for low fluorescence is that these surfaces didn’t allow bacteria to adhere on the surface and therefore grow further? If so, kindly clarify it in the manuscript.
3. Please provide statistical analysis for Fig 3A and how many replicates were performed?

**Minor concerns**

1) Introduction: The introduction provides a good overview but could benefit from a more detailed discussion of the current limitations of existing Ti and Ti alloys such as metal leaching in surrounding tissues and how this new biomaterial addresses these issues.

2) Methodology: While the general experimental setup is clear, some specific procedural details are missing. For instance, describe in more detail the protocol for in vitro antibacterial function, and Western Blot analysis including washing steps and transfer steps. Elaborate on labeling siRNA with FAM and how excess FAM was removed. Also, include the protocol for XPS analysis in the method section. Proliferation capacity of cells section mentions methylzan dye. Is that name right?

3) Figures and Tables: The figures and tables are generally clear, and well-prepared, however the captions of the figures lack explanation. I would highly advise to provide concise but comprehensive description in the captions. Also, the figures are not well explained in the manuscript text. For example, in Fig 6A and 7B, could you please explain the results in the manuscript by referring to the colored (or stained) regions in the images? It will be easier for a reader to follow the study better and quickly. In osteogenic differentiation on PEEK-pTAN-siRNA in vivo section (3.5): the reported results do not match for Tb.Sp in Fig6B. Kindly fix if it is a typo error.

4) Results and Discussion: The first paragraph seemed to be a repetition of text in introduction. I suggest authors to either remove it or modify it by adding additional relevant information.

5) References: Kindly check reference 6 and 39 do not seem to be right. Kindly make sure that these are correctly referenced.

6) Formatting issues: Kindly correct the following 2) include degree symbols for contact angle results in the text, b) siCKIP-1: the format is not correct for this molecule at various places, c) seems like something is missing in the 14th sentence in abstract; please complete it, d) the 3rd sentence under In vitro biocompatibility section (3.3) seems to have some text missing, kindly rephrase it, e) In osteogenic differentiation on PEEK-pTAN-siRNA in vivo section (3.5): Kindly insert hours after 12 in the second sentence, f) replace PEEK-pTAN with PEEK-pTAN-siRNA in the 6th sentence under Osteogenic differentiation on PEEK-pTAN-siRNA in vitro, g) the spelling of fluorescent in the 6th sentence under Immunofluorescence staining (2.13), h) fix the spelling of with in 2nd paragraph 2nd sentence under Results and discussion section (3.1).

**Recommendations**

Based on the above comments, I recommend that the manuscript be revised and resubmitted.

**Conclusion**

The manuscript presents significant findings, however, addressing the outlined concerns is crucial for ensuring the study's validity and impact. I look forward to reviewing the revised manuscript.
