# Supplementary material for: Enhanced bacteriostasis and osseointegrative properties of SiRNA-modified polyetheretherketone surface for implant applications
Source: PLoS One. 2024 Dec 5;19(12):e0314091. doi: 10.1371/journal.pone.0314091 (PMC11620434; doi:10.1371/journal.pone.0314091)
Supplement: Attachment — Submitted filename: Response to Reviwers.docx [file pone.0314091.s006.docx]

**Manuscript ID: PONE-D-24-26069**

**Title: Enhanced Bacteriostasis and Osseointegrative Properties of SiRNA-Modified Polyetheretherketone Surface for Implant Applications**

Dear editor:

On behalf of my co-authors, we thank you very much for giving us an opportunity to revise our manuscript, we appreciate the editor and reviewers very much for their positive and constructive comments. We have revised the whole manuscript according to the journal and reviewers' requirements and would like to re-submit it for your consideration. All revisions in the manuscript have been identified by using the red font. We have already addressed the comments raised by the reviewers. Below, we have listed our responses to each of the reviewers' comments, point by point.

Another noteworthy change is that we have altered the author order of the manuscript (in red font). Due to recent discussions within our research team and a more accurate reflection of individual contributions to this work, we propose the following revised author order: [Zhen Liu ^1,^ ^¶^, Libin Yang ^2,^ ^¶^, Yazhuo Ni ^3,&^, Keying Chen^3^, QiquanYan^3^, Zhiying Zhao^4^, Bo Xu^3^, Yaoyang Li^3^, Rui Li ^3,^ ^#a, &,*^Jianwen Li ^5,^ ^&,*^ ]. The reason for this change is that Zhen Liu has made significant contributions to the research and manuscript preparation, and we believe there is a reason to move her to the first position of co-first author. This decision is supported by the author team.

We hope the revised version of the manuscript is acceptable for publication in your journal now. Looking forward to hearing from you.

Yours sincerely,

Rui Li

Corresponding author: Rui Li

E-mail: kqxiufulirui@tmu.edu.cn

**ID: ACR-23-100**

**Reviewer #1:**

The manuscript addresses a significant topic in bone implantation and presents an efficient approach for modifying the surface of PEEK-implants, thus providing experimental evidence to support its clinical application. The methodology is sound and manuscript is clearly written, well-structured with clear research questions and a logical flow, however there are several areas including data interpretation, which require improvement to enhance the clarity and impact of the study.

Major Concerns

Comment 1: In Fig 1B, please provide Immunofluorescence images of control groups as well and explain the observation in the manuscript text. In Fig 1C, kindly mention whether you are reporting your surface roughness in Ra or Rq. Also, the AFM images seem to be hiding those numbers mentioned on top of each image. Additionally, please include more detailed analysis as to why you obtained those numbers and how they relate to the surface roughness of all three groups. For Fig 1D, you mentioned getting boost in N1s signal in PEEK-pTAN-siRNA surface. Could you provide expected atomic concentration in %? It would have been lot more helpful if the data was tabulated from XPS spectra.

Reply 1: We explained as follows: PEEK materials and polyphenol tannic acid coatings do not exhibit fluorescent properties, thus appearing as a black field under Confocal Laser Scanning Microscope. Refer to the following image.

In Fig 1C, we are reporting surface roughness in Ra. (The detailed statement in the manuscript is as follows: AFM results (Figure 1C) reveal PEEK sample as possessing a remarkably smooth surface (Ra=39.7nm). Conversely, the roughness of the PEEK-pTAN (Ra=48.1 nm) and PEEK-pTAN-siRNA groups(Ra=76.6 nm) increased, indicating that the surface of the modified PEEK was more was rougher.) The Hidden values have been marked in Fig 1C.

The supplementary results of Fig 1D are added to the supplementary material file in the form of table (Table S1). ( The detailed statement in the manuscript is as follows: The surface roughness of all three groups was analyzed by Nanoscope analysis software. The carbon to oxygen ratio (4.5) detected in the PEEK-pTAN group exceeded the theoretical C/O ratio (1.65) of pTAN(Table S1). This discrepancy suggests the presence of significant contamination from carbon-hydrogen compounds, as evidenced by the strong C 1s signal. In the PEEK-pTAN-siRNA group, a distinct nitrogen peak and a smaller phosphorus peak were clearly observed, accompanied by a boost in the N1s and P 2p contents to 12.84% and 1.1% (Table S1), respectively, confirming the successful loading of siRNA/PEI complex onto the PEEK-pTAN surface.)

Comment 2: In Fig 2 we see a lot less red fluorescence for PEEK-pTAN and PEEK-pTAN-siRNA groups. Do authors have other images showing better red fluorescence? Or the reason for low fluorescence is that these surfaces didn’t allow bacteria to adhere on the surface and therefore grow further? If so, kindly clarify it in the manuscript.

Reply 2: Red fluorescence represents dead bacteria. The amount of red fluorescence on the PEEK-pTAN and PEEK-pTAN-siRNA group surface decreased, which was caused by the weakening of adhesion ability after the death of bacteria on the sample surface. The detailed statement in the manuscript is as follows: In Figure 2, a low count of dead bacteria (red fluorescence) was observed in both the PEEK group and blank well. While dead bacteria (red fluorescence) of the PEEK-pTAN and PEEK-pTAN-siRNA group increased, indicating that pTAN coating enhanced the antibacterial activity of PEEK. At the same time, the amount of red fluorescence on the PEEK-pTAN and PEEK-pTAN-siRNA group surface decreased, which was caused by the weakening of adhesion ability after the death of bacteria on the sample surface (see Page 13, line342-347).

Comment 3: Please provide statistical analysis for Fig 3A and how many replicates were performed?

Reply 3: We conducted three repeated experiments, and the experimental data are supplemented in the annex (see the “CCK-8 Supplementary material.pdf”).

Minor concerns

Comment 1: Introduction: The introduction provides a good overview but could benefit from a more detailed discussion of the current limitations of existing Ti and Ti alloys such as metal leaching in surrounding tissues and how this new biomaterial addresses these issues.

Reply 1: We provided a brief description of Ti and Ti alloys in the introduction section, but still focused on the description of the Peek materials.

Comment 2: Methodology: While the general experimental setup is clear, some specific procedural details are missing. For instance, describe in more detail the protocol for in vitro antibacterial function, and Western Blot analysis including washing steps and transfer steps. Elaborate on labeling siRNA with FAM and how excess FAM was removed. Also, include the protocol for XPS analysis in the method section. Proliferation capacity of cells section mentions methylzan dye. Is that name right?

Reply 2: For the modification of the aforementioned issues, the following is the expression in the original text. We described in more detail the protocol for in vitro antibacterial function (see Page 6, line148-151), the Western Blot analysis including washing steps and transfer steps(see Page 8, line217-224). We added the details of labeling siRNA with FAM (see Page 5, line113) Proliferation capacity of cells section mentions formazan dye(see Page 7, line171).

Comment 3: Figures and Tables: The figures and tables are generally clear, and well-prepared, however the captions of the figures lack explanation. I would highly advise to provide concise but comprehensive description in the captions. Also, the figures are not well explained in the manuscript text. For example, in Fig 6A and 7B, could you please explain the results in the manuscript by referring to the colored (or stained) regions in the images? It will be easier for a reader to follow the study better and quickly. In osteogenic differentiation on PEEK-pTAN-siRNA in vivo section (3.5): the reported results do not match for Tb.Sp in Fig6B. Kindly fix if it is a typo error.

Reply 3: We have modified our text as advised (see Page 16, line438-440, 452-454).

Comment 4: Results and Discussion: The first paragraph seemed to be a repetition of text in introduction. I suggest authors to either remove it or modify it by adding additional relevant information.

Reply 4: We have removed the repetition of text in introduction.

Comment 5: References: Kindly check reference 6 and 39 do not seem to be right. Kindly make sure that these are correctly referenced.

Reply 5: We have rechecked reference 6 and 39 and recite the content in text (see Page 11, line292-294).

Comment 6:Formatting issues: Kindly correct the following 2) include degree symbols for contact angle results in the text, b) siCKIP-1: the format is not correct for this molecule at various places, c) seems like something is missing in the 14th sentence in abstract; please complete it, d) the 3rd sentence under In vitro biocompatibility section (3.3) seems to have some text missing, kindly rephrase it, e) In osteogenic differentiation on PEEK-pTAN-siRNA in vivo section (3.5): Kindly insert hours after 12 in the second sentence, f) replace PEEK-pTAN with PEEK-pTAN-siRNA in the 6th sentence under Osteogenic differentiation on PEEK-pTAN-siRNA in vitro, g) the spelling of fluorescent in the 6th sentence under Immunofluorescence staining (2.13), h) fix the spelling of with in 2nd paragraph 2nd sentence under Results and discussion section (3.1).

Reply 6: We have added the degree symbols for contact angle (see Page 23, line288-290) and modified the format of “siCKIP-1” (see Page 3, line83). We rephrase the 14th sentence of the abstract section (see Page 2, line38-39). The 3rd sentence under the section on In vitro biocompatibility has been rephrased (see Page 13, line357). In the osteogenic differentiation of PEEK pTAN siRNA in vivo, we have inserted hours after 12 in the second sentence (see Page 16, line436). Thank you very much for raising your writing question. We have replaced PEEK pTAN siRNA with PEEK pTAN siRNA in the sixth sentence of the osteogenic differentiation section of PEEK pTAN siRNA in vitro (see Page 14, line395). We have conducted spelling checks and made corrections (see Page 9, line238、see Page 11, line288).

**Reviewer #2:**

Comment: The title could be made more concise. For example, "Enhanced Bacteriostatic and Osseointegrative Properties of SiRNA-Modified Polyetheretherketone for Implant Applications." The abstract should include the main statistical tests used. In the introduction, a suggested improvement: "Therefore, identifying a new implant material with excellent biocompatibility and biomechanical properties is essential." Clarify the statistical methods used for data analysis in the Materials and Methods section. Ensure all figures in the Results section have complete and informative captions. Expand on the limitations of the study in the Discussion section, and ensure the conclusion highlights the study's significance and potential impact.

Reply: We appreciate you very much for your positive and constructive comments. We explained the different points as follows: The word 'surface' reflects the location where we modified the peek material, so we believe that the word 'surface' cannot be removed from the title. We have modified our text as advised in the Abstract (see Page 2, line 38-39). And the detailed descriptions have been added to the "Materials and Methods" and "Results and Discussion" sections (in red font).

Reviewer #3:

Comment: This is a nicely written paper with a comprehensive set of data including in vitro and in vivo studies. The authors provide detailed surface characterization results. Then, they provide comprehensive in vitro results with bacteria as well as mammalian cells. Moreover, the authors also provide comprehensive in vivo data showing promising results. A lot more can be done but the results presented are in a good manner.

Reply: We appreciate you very much for your positive and constructive comments. We hope the revised version of the manuscript is acceptable for publication in PLOS one.

**Journal Requirements:**

1.When submitting your revision, we need you to address these additional requirements. Please ensure that your manuscript meets PLOS ONE's style requirements, including those for file naming. The PLOS ONE style templates can be found at <https://journals.plos.org/plosone/s/file?id=wjVg/PLOSOne_formatting_sample_main_body.pdf> and

https://journals.plos.org/plosone/s/file?id=ba62/PLOSOne_formatting_sample_title_authors_affiliations.pdf

Reply: We revised the article according to the PLOS ONE's style requirements.

2. To comply with PLOS ONE submissions requirements, in your Methods section, please provide additional information regarding the experiments involving animals and ensure you have included details on methods of sacrifice and efforts to alleviate suffering.

Reply: We have included details on methods of sacrifice and efforts to alleviate suffering.

3. Thank you for stating in your Funding Statement: This study was supported by the Tianjin Key Medical Discipline (TJYXZDXK-038A) Construction Project. Please provide an amended statement that declares all the funding or sources of support (whether external or internal to your organization) received during this study, as detailed online in our guide for authors at http://journals.plos.org/plosone/s/submit-now. Please also include the statement “There was no additional external funding received for this study.” in your updated Funding Statement. Please include your amended Funding Statement within your cover letter. We will change the online submission form on your behalf.

Reply: We have amended Funding Statement, added the statement “There was no additional external funding received for this study.”

4. In the online submission form, you indicated that Supporting material is available from the corresponding author (kqxiufulirui@tmu.edu.cn) on reasonable request. All PLOS journals now require all data underlying the findings described in their manuscript to be freely available to other researchers, either a. In a public repository, b. Within the manuscript itself, or c. Uploaded as supplementary information. This policy applies to all data except where public deposition would breach compliance with the protocol approved by your research ethics board. If your data cannot be made publicly available for ethical or legal reasons (e.g., public availability would compromise patient privacy), please explain your reasons on resubmission and your exemption request will be escalated for approval.

Reply: Our manuscript to be freely available to other researchers which about supplementary information.

5. PLOS requires an ORCID iD for the corresponding author in Editorial Manager on papers submitted after December 6th, 2016. Please ensure that you have an ORCID iD and that it is validated in Editorial Manager. To do this, go to ‘Update my Information’ (in the upper left-hand corner of the main menu), and click on the Fetch/Validate link next to the ORCID field. This will take you to the ORCID site and allow you to create a new iD or authenticate a pre-existing iD in Editorial Manager. Please see the following video for instructions on linking an ORCID iD to your Editorial Manager account: https://www.youtube.com/watch?v=_xcclfuvtxQ.

Reply: We have added ORCID iD information to the identity of the corresponding author. ( Rui Li: 0000-0001-6407-7515)

6. Please include your full ethics statement in the ‘Methods’ section of your manuscript file. In your statement, please include the full name of the IRB or ethics committee who approved or waived your study, as well as whether or not you obtained informed written or verbal consent. If consent was waived for your study, please include this information in your statement as well.

Reply: We have added a complete ethical statement in the "Methods" section of the manuscript file.

7. Please remove your figures from within your manuscript file, leaving only the individual TIFF/EPS image files, uploaded separately. These will be automatically included in the reviewers’ PDF.

Reply: We have removed the charts from the manuscript file.

8. PLOS ONE now requires that authors provide the original uncropped and unadjusted images underlying all blot or gel results reported in a submission’s figures or Supporting Information files. This policy and the journal’s other requirements for blot/gel reporting and figure preparation are described in detail at https://journals.plos.org/plosone/s/figures#loc-blot-and-gel-reporting-requirements and https://journals.plos.org/plosone/s/figures#loc-preparing-figures-from-image-files. When you submit your revised manuscript, please ensure that your figures adhere fully to these guidelines and provide the original underlying images for all blot or gel data reported in your submission. See the following link for instructions on providing the original image data: <https://journals.plos.org/plosone/s/figures#loc-original-images-for-blots-and-gels>. In your cover letter, please note whether your blot/gel image data are in Supporting Information or posted at a public data repository, provide the repository URL if relevant, and provide specific details as to which raw blot/gel images, if any, are not available. Email us at plosone@plos.org if you have any questions.

Reply: We ensure that the included charts fully comply with the guidelines, and separately provide the original base images of all imprint or gel data submitted.

**PONE-D-24-26069R1**

**Journal Requirements:**

1. Thank you for stating the following financial disclosure: "This study was supported by the Tianjin Key Medical Discipline (TJYXZDXK-038A) Construction Project. There was no additional external funding received for this study" Please state what role the funders took in the study. If the funders had no role, please state: "The funders had no role in study design, data collection and analysis, decision to publish, or preparation of the manuscript." If this statement is not correct you must amend it as needed. Please include this amended Role of Funder statement in your cover letter; we will change the online submission form on your behalf.

Reply: This statement is not correct, we haved amend it. Corresponding author Rui Li serves as the project leader in the funding (Natural Science Foundation of Tianjin Municipality 18JCYBJC95500).

2. In the online submission form you indicate that your data is not available for proprietary reasons and have provided a contact point for accessing this data. Please note that your current contact point is a co-author on this manuscript. According to our Data Policy, the contact point must not be an author on the manuscript and must be an institutional contact, ideally not an individual. Please revise your data statement to a non-author institutional point of contact, such as a data access or ethics committee, and send this to us via return email. Please also include contact information for the third party organization, and please include the full citation of where the data can be found.

Reply: We have modified the visible range of the data, and all authors unanimously agree to disclose all data.

3. We note that you have indicated that there are restrictions to data sharing for this study. PLOS only allows data to be available upon request if there are legal or ethical restrictions on sharing data publicly. For more information on unacceptable data access restrictions, please see http://journals.plos.org/plosone/s/data-availability#loc-unacceptable-data-access-restrictions.Before we proceed with your manuscript, please address the following prompts: a) If there are ethical or legal restrictions on sharing a de-identified data set, please explain them in detail (e.g., data contain potentially identifying or sensitive patient information, data are owned by a third-party organization, etc.) and who has imposed them (e.g., a Research Ethics Committee or Institutional Review Board, etc.). Please also provide contact information for a data access committee, ethics committee, or other institutional body to which data requests may be sent. b) If there are no restrictions, please upload the minimal anonymized data set necessary to replicate your study findings to a stable, public repository and provide us with the relevant URLs, DOIs, or accession numbers. For a list of recommended repositories, please see https://journals.plos.org/plosone/s/recommended-repositories. You also have the option of uploading the data as Supporting Information files, but we would recommend depositing data directly to a data repository if possible. We will update your Data Availability statement on your behalf to reflect the information you provide."

Reply: All of the authors have confirmed that there are no restrictions to data sharing for this study, and we have optioned to upload the data as Supporting Information files.

4. “To comply with PLOS ONE submissions requirements, in your Methods section, please provide additional information regarding the experiments involving animals and ensure you have included details on methods of sacrifice. "We've returned your manuscript to your account. Please resolve these issues and resubmit your manuscript within 21 days. If you need more time, please email the journal office at plosone@plos.org. We are happy to grant extensions of up to one month past this due date. If we do not hear from you within 21 days, we will withdraw your manuscript.

Please log on to PLOS Editorial Manager at https://www.editorialmanager.com/pone/ to access your manuscript. You will find your manuscript in the 'Submissions Sent Back to Author' link under the New Submissions menu. Be sure to remove your previous manuscript file if you are uploading a new file in response to these requests. After you've made the changes requested above, please be sure to view and approve the revised PDF after rebuilding the PDF to complete the resubmission process.

Reply: We have supplemented the content again (in red font).
